# A Bayes Decision Rule to Assist Policymakers during a Pandemic

**DOI:** 10.3390/healthcare9081023

**Published:** 2021-08-09

**Authors:** Kang-Hua Cao, Paul Damien, Chi-Keung Woo, Jay Zarnikau

**Affiliations:** 1 Department of Economics, Hong Kong Baptist University, Hong Kong, China; kanghuacao@hkbu.edu.hk; 2 Department of Information, Risk and Operations Management, McCombs School of Business, University of Texas in Austin, Austin, TX 78712, USA; 3 Department of Asian and Policy Studies, The Education University of Hong Kong, Hong Kong, China; chiwoo@eduhk.hk; 4 Department of Economics, University of Texas in Austin, Austin, TX 78712, USA; jayz@utexas.edu

**Keywords:** Bayesian inference, decisions, employment, mortality rates, net benefit, sensitivity analysis, D10, D60, I10, I18

## Abstract

A new decision rule based on net benefit per capita is proposed and exemplified with the aim of assisting policymakers in deciding whether to lockdown or reopen an economy—fully or partially—amidst a pandemic. Bayesian econometric models using Markov chain Monte Carlo algorithms are used to quantify this rule, which is illustrated via several sensitivity analyses. While we use COVID-19 data from the United States to demonstrate the ideas, our approach is invariant to the choice of pandemic and/or country. The actions suggested by our decision rule are consistent with the closing and reopening of the economies made by policymakers in Florida, Texas, and New York; these states were selected to exemplify the methodology since they capture the broad spectrum of COVID-19 outcomes in the U.S.

## 1. Introduction

The aim of this research is to develop a simple and useful decision rule to better assist policymakers amidst a pandemic so they can decide whether to open all or parts of an economy. COVID-19 data from the U.S. are used to illustrate the rule. We employ a Bayesian simulation-based decision analysis approach. 

Our contributions are four-fold. First, decision rules are generally predicated on uncertain events. Using modern Bayesian methodological tools [1], we quantify our decision rule via probability distributions. This approach in the decision analysis literature is also in line with what [2] advocate. They recommend that the principles of decision analysis should play a more prominent role in actual political, decision-making processes. Additionally, they also note that Bayesian statistics should be better employed to support the public understanding of societal issues. Second, unlike other recent pandemics, COVID-19 presents unique challenges to policymakers; reliable data are one of those challenges. We note at the outset that our approach is invariant to the choice of country or pandemic type. Third, the methodology lends itself to performing several “what if” analyses based on credible input assumptions that different policymakers may want to evaluate. Finally, while this paper’s decision rule (stated below) may be viewed as the outcome of a cost–benefit analysis, its modelling is innovative, based on an econometric setup that considers some of the key aspects of pandemics. These include infection and death rates, employment and income impacts of pandemic suppression, and treatment and fatality costs of a pandemic.

Within the context of pandemics, we now briefly describe the intuition underlying our contributions. We define the phrase, “decremental suppression” to mean reopening the economy—partially or fully— after it has been closed due to public health reasons. Thus, the aim is to develop a decision rule for a government to determine on day *d* if decremental suppression should occur given the information available on that day. This probabilistic rule is formulated by first defining the net benefit per capita based on a standard benefit–cost analysis:

Net benefit per capita = per capita income increase − per capita fatality cost increase − per capita medical cost increase

For convenience, we denote the left-hand side of the above formulation ΔNB to mean change in household net benefit. Then, our main recommendation to public officials may be stated as a Decision Rule: *decremental suppression should occur when* ΔNB > 0 *and P*(ΔNB > 0) ≥ *T*. The choice of the threshold probability *T* is deliberately left unspecified since it is, typically, made by an individual decision-maker or a body collective, i.e., the selection of *T* is a standard of judgment that can only be arrived at subjectively, based on the analytics. Intuitively, suppose *T* > 0.9; then, a decremental suppression decision can be thought of as having a very low risk. Later, via several sensitivity analyses, we demonstrate various judgments about *T* for the COVID-19 pandemic in the U.S. The important point to be made is that, given the data, we can quantify the decision rule via probability distributions.

To develop the probability distributions, we propose and implement two econometric specifications: one for consumption and another for fatality rate. The output from the former quantifies the benefit element while the latter estimates the cost elements via the pandemic’s impact on the health of a population. 

Our empirical work showcases our methodology for COVID-19, which was declared a pandemic by WHO in 2020 (https://www.who.int/emergencies/diseases/novel-coronavirus-2019/situation-reports, accessed on 10 July 2020). While COVID-19′s spread had peaked in China by mid-February 2020, it sharply surged in March 2020 in the U.S. In response, federal, state, and local officials in the U.S. adopted suppression measures to curb economic activities that accelerate the spread of a viral disease [3,4,5], including shelter-in-place, social distancing, mask wearing, travel restrictions, business and school shutdowns, etc. Agüero and Beleche [6] discussed the merits of good hygiene during the H1N1 pandemic in Mexico, while [7] compared social distancing measures and their effects on dealing with pandemics. An interesting result was reported in [8], who found that, to better control epidemics, treatment should be administered in regions with *lower* infections. They noted that trying to equalize infections in two interconnected regions where one is high and the other low is the worst possible strategy.

Sands et al. [9] argued that macroeconomic forecasting methodologies should incorporate the impact of pandemics in order to better model the negative consequences of contagious diseases on the world economy. Karlsson et al. [10] modelled the impact of mortality on earnings, capital returns, and populations living in poorhouse counties in Sweden during the Spanish Flu.

In the realm of decision and risk analysis, [11,12] considered the economy-wide impacts of an influenza pandemic using a large-scale modelling approach. They utilized the interoperability input–output (I–O) model to analyse the impacts of an influenza pandemic. The I–O framework captures the interactions among different sectors in an economy and highlights the impacts on workforce disruption. 

The cost of suppressing economic activity in this pandemic has received attention and is ongoing. Prager et al. [13], using a computable general equilibrium model, found that, without vaccines, a loss of $25.4 billion in GDP may occur due to a pandemic influenza outbreak in the U.S. Scherbina [14] estimated that COVID-19′s total economic cost without suppression in the US exceeds $9 trillion, comprising medical cost, value of lost productivity, and fatality cost based on value-of-statistical-life (VSL). The total cost estimate for a 78-week suppression period is, however, much lower at $15.8 billion. Ugarov [15] considered three policy approaches to address COVID-19′s spread: (1) do nothing, (2) keep the number of new cases via non-pharmaceutical suppression at the maximum of health care capacity, and (3) reduce the number of new cases via non-pharmaceutical suppression plus extensive testing and case isolation. Ugarov [15] found that (3) results in the lowest cost of $5.4 trillion, comprising VSL, direct medical costs, employment losses, and mandatory shutdown costs. Greenstone and Nigam [16] reported that the total benefit of social distancing based on VSL is roughly $8 trillion. Pearson et al. [17] found that test-then-vaccinate improved health care while reducing costs when administering dengue vaccines. In the interests of brevity, we omit other cost studies similar to the above. 

Our goal is to better model the impact of a pandemic’s decremental suppression on the net benefit per capita. For COVID-19, the importance of such a modelling effort is underscored (a) by state government plans announced in late April 2020 to ease suppression in May 2020, thereby reversing the sharp spike in unemployment triggered by the pandemic (https://edition.cnn.com/2020/04/30/economy/unemployment-benefits-coronavirus/index.html, accessed on 10 July 2020), and (b) by decisions faced by policymakers during the H1N1 pandemic based on the economic costs of suppression [2,18,19,20].

Official data show that state-by-state variations in COVID-19′s impact is substantial. We demonstrate our methodology using Florida, New York, and Texas since, based on publicly available reports and data, they capture the spectrum of COVID-19 outcomes across the U.S.

The rest of the paper is organized as follows. The next section details the econometrics underlying our estimation of the benefit component. This is followed by the description of medical and fatality costs in Section 2 and Section 3, respectively. An illustrative analysis leading to the calculation of the decision rule is described in Section 4. A discussion in Section 5 concludes the paper.

## 2. Benefit Component and Its Econometric Representation

How should one model per capita income increase? A regression model for income, conditioned on appropriate exogenous variables, could be constructed. The difficulty lies in the time scale. Recent experience shows that the pandemic context is better served by using weekly data since mortality and unemployment data evolve weekly. Moreover, state-level income data is, typically, reported quarterly and often not at the same time. Converting such data to weekly numbers, while possible, is inadvisable since the resulting estimation of model parameters is exposed to considerable added noise in the data. Therefore, we use weekly percent employed as the response variable to arrive at income. Below, we arrive at weekly income by modelling weekly employment that is assumed to depend on the pandemic’s weekly infection rate. Additionally, the periodicity and availability of percent employed is the most granular and, hence, reliable. The predicted weekly employment can be used to closely approximate changes to weekly income using most recent, publicly available, median and/or per capita income figures. The labour input might change from week to week due to paid leave. Such a leave may be more likely to occur during COVID-19 due to self-quarantine and sick leave measures. It could be argued that these factors may not be captured in the employment variable. However, while labour usage may vary weekly due to paid leave, the employment data measure workers who are on the payroll of companies. By contrast, self-quarantine and layoff due to COVID-19 suppression reduce employment and therefore income.

With *t* denoting week, for *t =* 1, …, *T*, consider the following econometric specification for employment *E_t_* (the dependent variable) within a state in the U.S.:

*E_t_* = *α*_0_ + *α*_1_*c_t_* + *α*_2_*d_t_*+ *ϕ*_1_*E_t−_*_1_ + … + *ϕ_p_E_t−l_* + *ε_t_*. (1)

In Equation (1), *c_t_* is the total number of confirmed pandemic-related infections divided by the total number tested: a priori, ceteris paribus, we would expect its coefficient, *α*_1,_ to be negative, i.e., an increase in the number of infected cases due to decremental suppression could lead to a fall in income. (Although many may work from home, their income likely declines due to employment and travel restrictions, such as shelter-in-place and social distancing. Note, also, that employment data exclude those that are not in the labour force.) The variable *d_t_* is a regime shift dummy variable. We purposely use data from 2007 so that we can include the impact of the 2008 recession. The economic downturn in the current pandemic most closely resembles that time frame. This is one of the strengths of the Bayesian approach, for it allows the model parameters to *learn* from past data. Hence, having a dummy variable to capture the two regime shifts in the economy is useful; this variable is coded 1 during the shifts and zero elsewhere. The employment rate time series *E_t_* is likely to be autocorrelated; hence, we include an AR(*l*) process in (1). To determine the optimal lag *l* for each state, in our analysis, we use a model selection procedure based on Bayesian posterior probabilities. It is worth emphasizing that this order could differ across states, depending on how a pandemic affects a state. The error term, *ε_t_*, is normally distributed with mean zero and unknown variance *σ^2^*. Following the selection of *l* and given future values of *c_t_*, we can obtain the corresponding predictive distribution for *E_t_* using Markov chain Monte Carlo (MCMC) methods [21,22,23]. Since the methodology is well-known, we omit details.

To complete the Bayesian construction of Equation (1), proper prior distributions on all of the unknown, random parameters are needed. Barnett et al. [23] noted that stationarity requires −1 ≤ *ϕ*_1_ ≤ 1; hence, a uniform prior distribution on [−1, 1] was used. If necessary, the same proper prior may be used for all of the other autoregressive parameters. For the variance *σ*^2^, an inverse-gamma prior with hyperparameter values equal to 0.01 was used; *α*_0_, *α*_1_, and *α*_2_ were assigned normal distributions with mean 0 and variance 100. All of the hyperparameter prior choices reflect diffuse beliefs.

It is possible, in principle, to construct subjective, informative priors for the hyperparameters. However, this is quite involved; moreover, we wanted the data to dictate the inference as much as possible, thereby mitigating bias in the empirical results. Another reason for assigning large variances to the model parameters is that it lessens the dependency on the prior choices.

Equation (1) is the benefits component of our pandemic welfare model. We prefer this single equation model as we model the percent employed in each state. Later, we discuss other modelling possibilities. It would appear that we assume that aggregate consumption is purely a function of income. This is a fair criticism since government-mandated shutting down of an economy could also potentially impact aggregate supply. However, we argue that the downward shock to demand in the current pandemic was and still is very large; this, we concede, has compromised the supply of many goods and services. However, aggregate supply and supply chains were never entirely shut down since most essentials were and are still very much in demand.

In addition to data difficulties, issues of the type discussed above are other reasons as to why we do not model income or consumption directly. 

## 3. Pandemic Costs and Their Econometric Framework

During a pandemic, the cost component in calculating NB comprises expected medical and expected mortality costs. The former cost includes three elements: (1) an uninfected person with symptoms, (2) an infected patient who recovers, and (3) an infected person who despite medical treatment eventually dies. Details of the calculation of all of the costs are described later. Here, we merely note that a critical input needed in those calculations is the predicted death (also known as fatality) rate that depends on the infection rate, *c_t_*. In the following, we model the fatality rate exactly as *E_t_* in Equation (1) with one noteworthy difference. In Equation (1), we used weekly employment data starting in 2007; hence, the COVID-19 infection rate exogenous variable, *c_t_*_,_ is recorded as zero until sometime in 2020 when data for it began to be collected in the U.S. Thus, for any COVID-19 mortality rate estimation/prediction in the U.S., state-level data only begins in late February or early March of 2020. Hence, a weekly mortality model suffers from sample size limitations. Using observed daily data obviates this issue. This, of course, does not adversely impact the eventual calculation of *P*(ΔNB) in our decision rule.

Let *w_t_* denote the death rate on day *t* in a given state where *w_t_* is defined as the total number of deaths on day *t* divided by the cumulative number of confirmed cases on day *t −* 7, with *t =* 8, *…*, *T*. This ratio is consistent with what is reported by government/health agencies; additionally, fatalities on any given day result from past infections since it takes time for the virus to turn lethal in some individuals. 

We use the exogenous variable *c_t_* as in Equation (1) but replace the weekly infection rates with daily rates instead. Thus, *c_t_* links the benefit and cost components of our econometric models. Given the serial correlation in *w_t_*, we also add a general autoregressive process, AR(*m*), to model each state’s fatality time series and write:

*w_t_* = *γ*_0_ + *γ*_1_*c_t_* + *ϕ*_1_*w_t−_*_1_ + … + *ϕ_p_w_t−m_* + *ε_t_*, (2)

where *ε_t_* is normally distributed with mean zero and unknown variance *σ*^2^. The rest of the modelling process, including prior distribution selections, resembles what was detailed earlier. Note that the rationale for associating the death rate with the infection rate is because a COVID-19 related death cannot occur unless the dead person is an infected patient in the first place. Moreover, deaths on a given day can occur for COVID-19 patients infected during prior days, which can straddle adjacent months. For example, a patient may survive many days after first being hooked-up to a respirator and then moved to an ICU ward. Hence, we link the death rate with the infection rate based on cumulative cases.

Taken together, Equations (1) and (2) constitute our pandemic econometric models. However, to reiterate, the specific forms for the relationships between the endogenous and exogenous variables in (1) and (2) could be changed, either by adding different exogenous factors and/or different representations such as panel data models. However, such changes do not change the overall methodological framework. As long as (Bayesian) predictive income and cost distributions are available via Equations (1) and (2), the rule *P*(ΔNB > 0) *≥ T* can be adequately quantified.

## 4. Empirical and Decision Analysis

The aim is to obtain a probability distribution for ΔNB. To reach our goal, we need to complete the analysis of Equations (1) and (2).

To illustrate our ideas, we use data from Florida (FL), New York (NY), and Texas (TX). NY was hit the hardest. FL was one of the first states to recognize that COVID-19 affected seniors (greater than 65 years of age) the most and took measures to curb the fatality rate in that group, leading to markedly fewer casualties. TX lies somewhere in the middle and is most representative of several states.

To model Equation (1), our weekly percent employed data starting in January 2007 and ending on 30 April 2020. (The weekly percent employed data are available at www.bls.gov accessed on 10 July 2020). As noted earlier, we chose to include the recession years 2008 through early 2010. This is because that time frame covers a sharp downward trend in economic activity, somewhat consistent with what is going on now. Even though the H1N1 pandemic started in 2009, its impact was quickly brought under control by the end of that year due to new vaccines and the mutation of the virus itself into milder forms. As such, despite its negative impact on the economy, H1N1 did not materially affect the U.S. economy as much as the 2008 financial crisis. There is “information” in the numbers from that crisis that is worth using in the current calamity. (We also performed our analysis without the 2008–2011 data. While the overall conclusions were qualitatively similar when starting with data from 2012, the variability in the parameter estimates and the predictive distributions were much smaller. We felt that they underestimated the truth of what is going on currently. Therefore, we used the larger dataset to model employment in Equation (1).) Hence, we used a dummy variable in Equation (1) to capture the 2008 and 2020 regime shifts in the employment time series.

To model Equation (2), we used data from the day that the first death was recorded in each state; hence, the start date varies for the three states, but the end date was set to 30 June 2020 for all three states to reflect the latest data available at the time of our writing. (The daily COVID-19 data were provided by the COVID Tracking Project (https://covidtracking.com/) launched from *The Atlantic*.)

**Figure 1:**
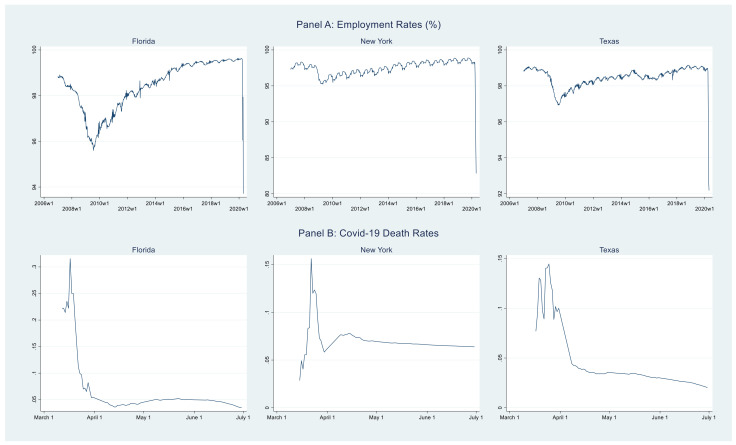
Weekly employment rates (2006–2020) and daily COVID-19 death rates (2020) by states.

**Figure 2:**
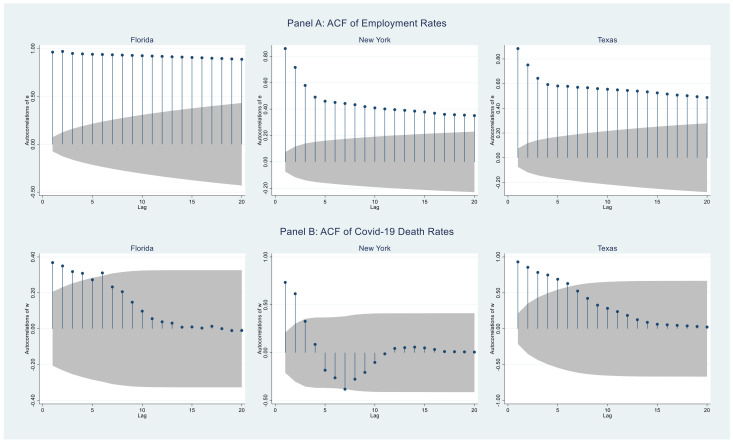
ACF plots for the employment and death rate series by state.

Consider Figure 1. The top (bottom) three panels show the weekly (daily) time series for percent employed (death rate) for the three states. To assess the autocorrelation in the data, consider Figure 2. The top (bottom) three panels show the ACF plots for the employment (death rate) series from Figure 1. Clearly, as expected, there is considerable autocorrelation in these data.

In the following, we provide details only for the Florida model since the other two states were similarly handled. However, where appropriate, we contrast the key takeaways for all three states via tables and/or graphs.

*Software and convergence notes*: STATA was employed to perform the calculations, where we set the number of iterations to 20,000 with a burn-in of 10,000 in each MCMC chain. The algorithms converged quite quickly with two chains, but nonetheless, we tested them with multiple MCMC chains. We followed the recommendations on the convergence diagnostics discussed, for example, in [1,24]; these diagnostics are provided in a Supplementary Materials Table S1 and Figures S1–S4 for the FL model since the NY and TX models are similar. All results are based on two MCMC chains.

### 4.1. Analysis of Equation (1)

The first step is to determine how many lags to use in Equation (1). The PACF values (not shown here) corresponding to the ACF plots in Figure 2 suggested that the model might require up to seven lags. Therefore, we used a Bayesian model selection procedure that calculates the marginal likelihood for seven models, where each model also includes the exogenous variable, *c_t_*, and the dummy variable *d_t_*. 

From the first column of Table 1, it is evident that a model with four lags is best for the Florida employment data. That is, in Equation (1), *l* = 4.

Now, for any given infection rate and the value of *E_t_* on 30 April 2020, we can obtain the predictive distributions *E_t+_*_1_ after estimating the following:

*E_t_ = α_0_ + α_*1*_c_t_ + α_*2*_d_t_ + ϕ_*1*_E_t−*1*_ + ϕ_*2*_E_t−*2*_ + ϕ_3_E_t−*3*_ + ϕ_*4*_E_t−*4*_ + ε_t_*. (3)

Finally, for each household, under each infection rate assumption, we can obtain the corresponding *annual* personal income distribution from the *E_t+_*_1_ distribution since the income distribution is merely a function of *E_t+_*_1_. To obtain this distribution, we use publicly available 2019 per capita personal income from Florida ($52,426). In a “what if” analysis, we also consider the unemployment benefit provided by the U.S. government and adjust the 2019 Florida income to $58,371; this type of adjustment stems from the Coronavirus Aid, Relief, and Economic Security (CARES) Act. To convert the percent employed predictions into annual figures, we multiply *E_t+_*_1_/100 by the income measures. 

The key point in all of the above is that we can obtain the probability distribution for the predicted personal income since, at each MCMC iteration, values from these distributions are merely functions of the samples from the posterior distributions of the parameters given in Equation (3).

The summaries—mean, standard deviations, and 95% highest probability density (HPD) intervals—of the posterior distributions of the regression parameters, *α*_0_, *α*_1_, *α*_2_, *ϕ*_1_, *ϕ*_2_, *ϕ*_3_, and *ϕ*_4_ and the variance *σ*^2^ are shown in Panel A of Table 2. The parameters *α*_1_ and *α*_2_, corresponding to infection rate and the regime change dummy variable, are of particular interest. Their 95% HPD intervals cover negative values. That is, when weekly infection rate increases, ceteris paribus, weekly employment declines. Likewise, both in the 2008 recession and the 2020 pandemic periods, employment declines, as evidenced by the HPD for *α*_2_. This “learning from experience” (past data) is a strength of the Bayesian approach.

**Figure 3:**
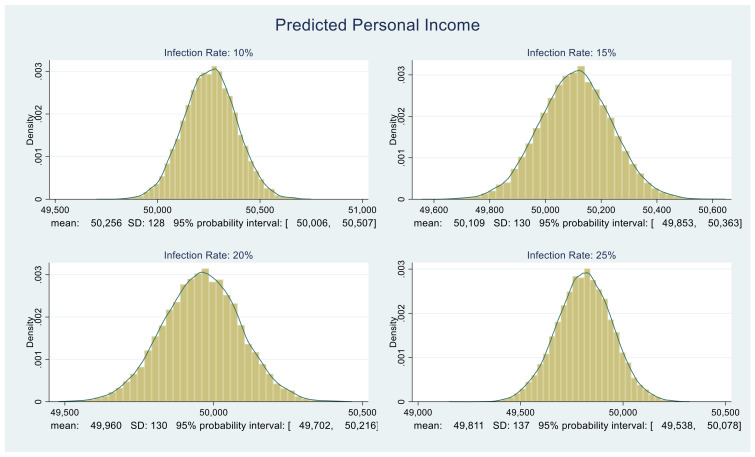
Predictive distributions of Florida unadjusted personal income in dollars.

**Figure 4:**
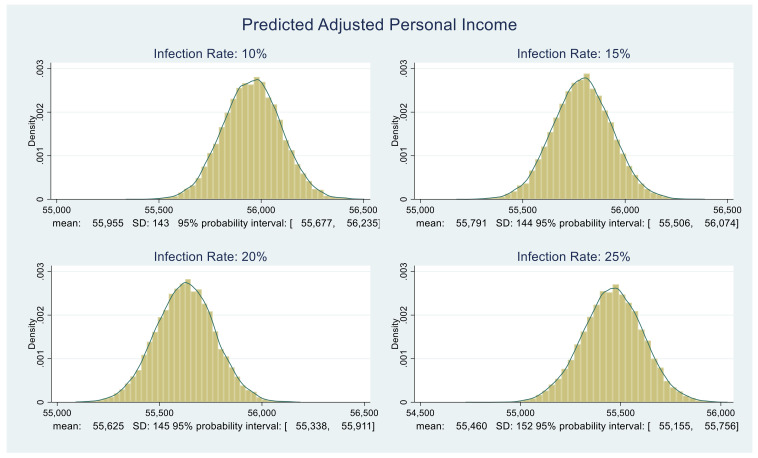
Predictive distributions of Florida adjusted personal income in dollars.

Consider Figure 3 and Figure 4. For four different values of infection rate, 10%, 15%, 20%, and 25%, we plot the corresponding predictive distributions of the personal income and the CARES Act adjusted personal income, respectively. The corresponding means, standard deviations, and 95% probability intervals appear below each graph.

At this stage, we quantified the benefit component of the NB calculation. In essence, we quantified the benefit for an individual within a household. To convert the values in Figure 3 and Figure 4 into household income figures, we could multiply each value in the distribution by the average household size in Florida. We prefer not to do this since it might inflate benefits. There is considerable anecdotal and relevant official evidence that at least one employed person in many homes was laid off, furloughed, or forced to work part-time. 

### 4.2. Analysis of Equation (2)

The predictive distribution of *w_t_* from Equation (2) serves as a key input in calculating the second component of NB, namely expected medical cost and fatality cost. We tackle each in turn. (These costs are for an individual. For a household, we could once again multiply the final cost values by the average size of households in a state, similar to what that for the benefit values.)

*Medical and fatality costs.* There are three types of medical costs due to decremental suppression: (1) Δ*λ_S_*_1_ for an uninfected person with COVID-19 symptoms (e.g., fever, coughing, and sore throat), (2) Δ*λ_S_*_2_ for an infected patient who recovers, and (3) Δ*λ_S_*_3_ for an infected person who despite medical treatment eventually dies. We formulate the cost equation for each type, followed by Δ*λ_D_* for the expected fatality cost increase due to decremental suppression.

(1) On day *t*, let Δ*λ_S_*_1_= (1 − *w_t_ c_t_*) *C_S_*_1_ − (1 − *w*_0_ *c*_0_) *C_S_*_1_ = (*w*_0_ *c*_0_ − *w_t_ c_t_*) *C_S_*_1_. We assume *C_S_*_1_ = $2000 per uninfected person for testing and treatment; *w*_0_ and *c*_0_ are 3.48% and 7.84%, the respective death and infection rates on 30 June 2020 in Florida.
(2) On day *t*, let Δ*λ_S_*_2_ = (1 − *w_t_ c_t_*) *C_S_*_2_ − (1 − *w*_0_ *c*_0_) *C_S_*_2_ = (*w*_0_ *c*_0_ − *w_t_ c_t_*) *C_S_*_2_. We assume *C_S_*_2_ = $15,000 for an infected person’s treatment that includes hospitalization. These cost estimates are consistent with that in [25]; they are linear in the infection rate, and we adjusted the cost with the infection rate.
(3) On day *t*, let Δ*λ_S_*_3_ = (1 − *w_t_ c_t_*) *C_S_*_3_ − (1 − *w*_0_ *c*_0_) *C_S_*_3_ = (*w*_0_ *c*_0_ − *w_t_ c_t_*) *C_S_*_3_. We assume *C_S_*_2_ = *C_S_*_3_ since we do not accurately know whether a recovered patient has longer hospitalization and more intense treatment than a dead patient.
(4) Let the change in *expected fatality cost* on day *t* be Δ*λ_D_* = (*w_t_ c_t_* − *w*_0_ *c*_0_) *C_D_*. We let *C_D_* = $7 million, the age-dependent VSL-based fatality cost.

Hence*,* decremental suppression’s *total cost* on day *t* is (1) + (2) + (3) + (4) = (*w_t_ c_t_* − *w*_0_ *c*_0_) (*C_D_* − *C_S_*_1_ − *C_S_*_2_ − *C_S_*_3_) = (*w_t_ c_t_* − *w*_0_ *c*_0_) × (7 million − 2K − 15K − 15K) = (*w_t_ c_t_* − *w*_0_ *c*_0_) × $6,968,000. It highlights (i) that a COVID-19 patient’s fatality cost dominates medical costs when calculating ΔNB’s cost component; (ii) that imprecision in the medical cost assumptions is unlikely to materially alter decremental suppression’s decision by a state; and (iii) that excluding decremental suppression’s fatality cost impact would vastly, though erroneously, overstate ΔNB.

Now, to complete the total cost calculation, we need estimates of the endogenous quantity *w_t_*. The estimation and simulation of Equation (2) is similar to Equation (1) (equivalently Equation (3)). For Florida, the only difference is that, instead of four lags of the dependent variable in the employment model, the Bayesian model selection procedure requires two lags of *w_t_*; see second column in Table 1. We omit other details, except to note that the posterior summaries for the death rate model for Florida that appears in Panel B of Table 2. In this table, consider the 95% HPD interval for the parameter, *γ*_1_, corresponding to daily infection rates. This interval contains zero. This is consistent with the time series plot of the death rate values shown in Figure 1 as well as media/governmental reports. During the early-to-middle stages of the pandemic, while social distancing and other mitigating measures were beginning to be enforced, death rates were on the rise with increasing infections. However, once the measures began to take effect and Florida aggressively quarantined the most vulnerable groups, death rates declined even though infections were still on the rise, albeit at a decreasing rate. While forecasting is not the primary aim of this paper, nonetheless, we tested the fatality model’s out-of-sample performance, which is the gold standard in time-series models. For New York, the one-week ahead out-of-sample root mean square error was less than eight percent, while for Florida and Texas, they were less than five percent. 

**Figure 5:**
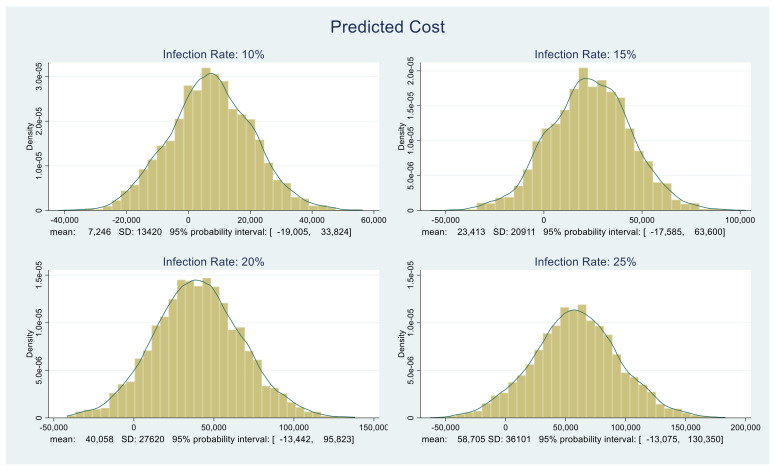
Predictive distributions of medical and fatality costs in dollars.

The resulting cost distributions with appropriate summaries under each of the four infection rates, 10%, 15%, 20%, and 25%, are shown in Figure 5. Depending on the infection rate, costs may be negative. This is to be expected since we drew random values from various probability distributions; hence, for certain combinations of the components comprising the overall cost distribution (detailed above), the costs could be negative. The important point here is costs are and should be an increasing function of infection rates. The predictive distributions shown in Figure 5 confirm this.

### 4.3. Calculating ΔNB and P(ΔNB > 0)

**Figure 6:**
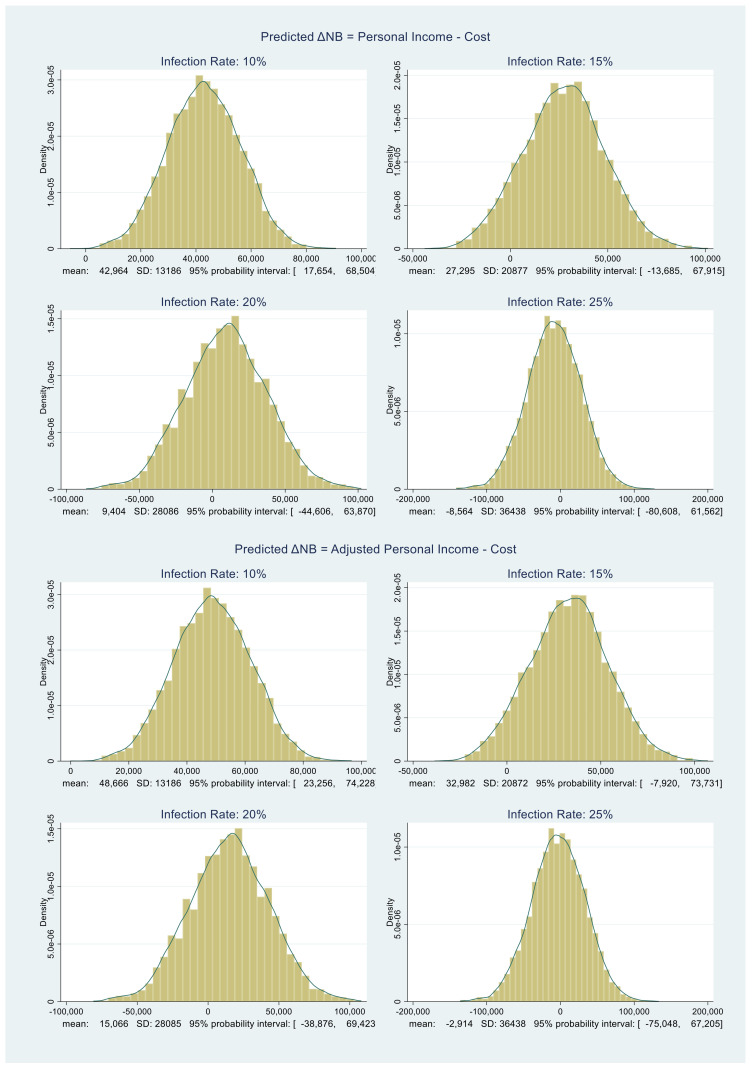
Predictive distributions of Florida ΔNB in dollars.

All that remains is to obtain the distribution of ΔNB. The benefit distributions in Figure 3 and Figure 4 and the cost distributions in Figure 5 are approximately normal. We compute their MCMC-based means and standard deviations. Using those, under each infection rate assumption, we randomly draw 5000 samples from the corresponding benefit and cost distributions and subtract them. The resulting ΔNB distributions are shown in Figure 6. 

Consider Table 3, which provides the summary statistics for the distributions in Figure 6. We are now ready to quantify our decision rule for Florida. 

*The Personal Income Probabilities*: From the last row of Table 3 under Personal Income, when ΔNB > 0, *P*(ΔNB > 0) is equal to 0.9998, 0.8996, 0.6344, and 0.4104 corresponding to infection rates 10%, 15%, 20%, and 25%, respectively. 

*The Adjusted Personal Income Probabilities*: From the last row of Table 3 under Adjusted Personal Income, *P*(ΔNB > 0) is equal to 0.9998, 0.9394, 0.7072, and 0.4738. 

Table 4 and Table 5 provide similar summaries for New York and Texas, respectively. We now turn to the policy discussion of the above scenarios.

### 4.4. Sensitivity Analyses via Probability Plots 

Once the samples from the posterior distributions of the parameters are saved, then it is straightforward to implement as many sensitivity analyses as one wants. The first part of our decision rule requires that we consider only those scenarios for which ΔNB > 0. Given this condition, we then examine the following scenarios for FL, NY, and TX. The metrics used are per capita personal income (Bureau of Economic Analysis), median household income (Census), per capita income (Census), and the CARES Act adjusted version of the three measures. 

As shown earlier, the infection rates considered in the sensitivity analysis are 10%, 15%, 20%, and 25%. It should be noted that, in the sample period used, the largest infection rate in Florida was 12.4%. Thus, our analysis veers in the direction of extreme caution by performing a sensitivity analysis with very large infection rates. Likewise, for Texas, the largest infection rate was 9.6%. New York reached an infection rate of 38.8%. However, as we see later on, based on our decision rule, even at 25%, a decision to segment and isolate at least some population groups (e.g., elderly) would have been a prudent choice for New York.

Another reason for varying the exogenous input infection rates is to indirectly assess the impact of non-pharmaceutical interventions (NPIs) such as lockdowns, partial shutdowns, social distancing, etc. on death rates and employment. At least 30 studies have shown that NPIs have been *ineffective* (https://inproportion2.talkigy.com/do_lockdowns_work_2021-01-15.html, accessed on 10 July 2020). Be that as it may, suppose one believes masks could mitigate infection rates, whence death rates, then, ceteris paribus, it stands to reason that higher infection rates might be the result of poor NPI protocols. Conversely, effective use of NPIs might lead to lower mortality. (We note that the jury is clearly out on the value of NPIs in this pandemic; we discuss this in the final section.) For our purposes, performing a “what if” analysis with different infection rates serves to illustrate the overall methodology and its value to policymakers, notwithstanding one’s beliefs about the efficacy of NPIs.

Regardless of which income measure one uses, our decision rule states: If *P*(ΔNB > 0) *≥ T*, decremental suppression should occur.

**Figure 7:**
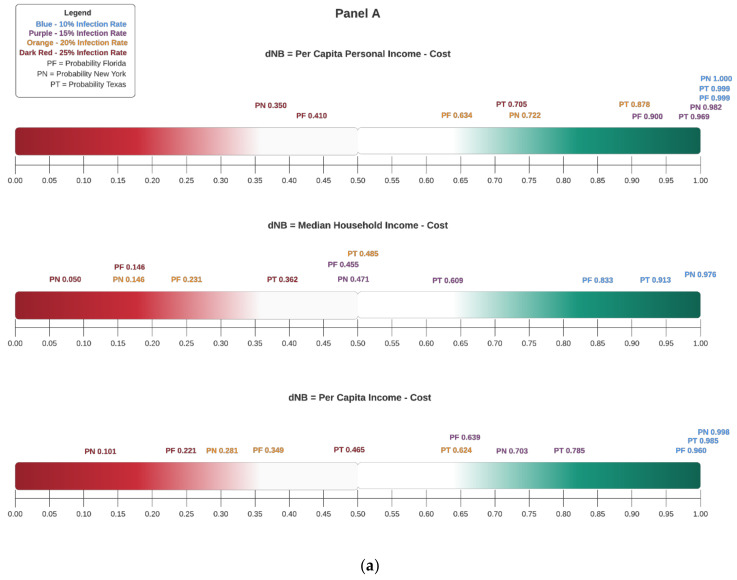

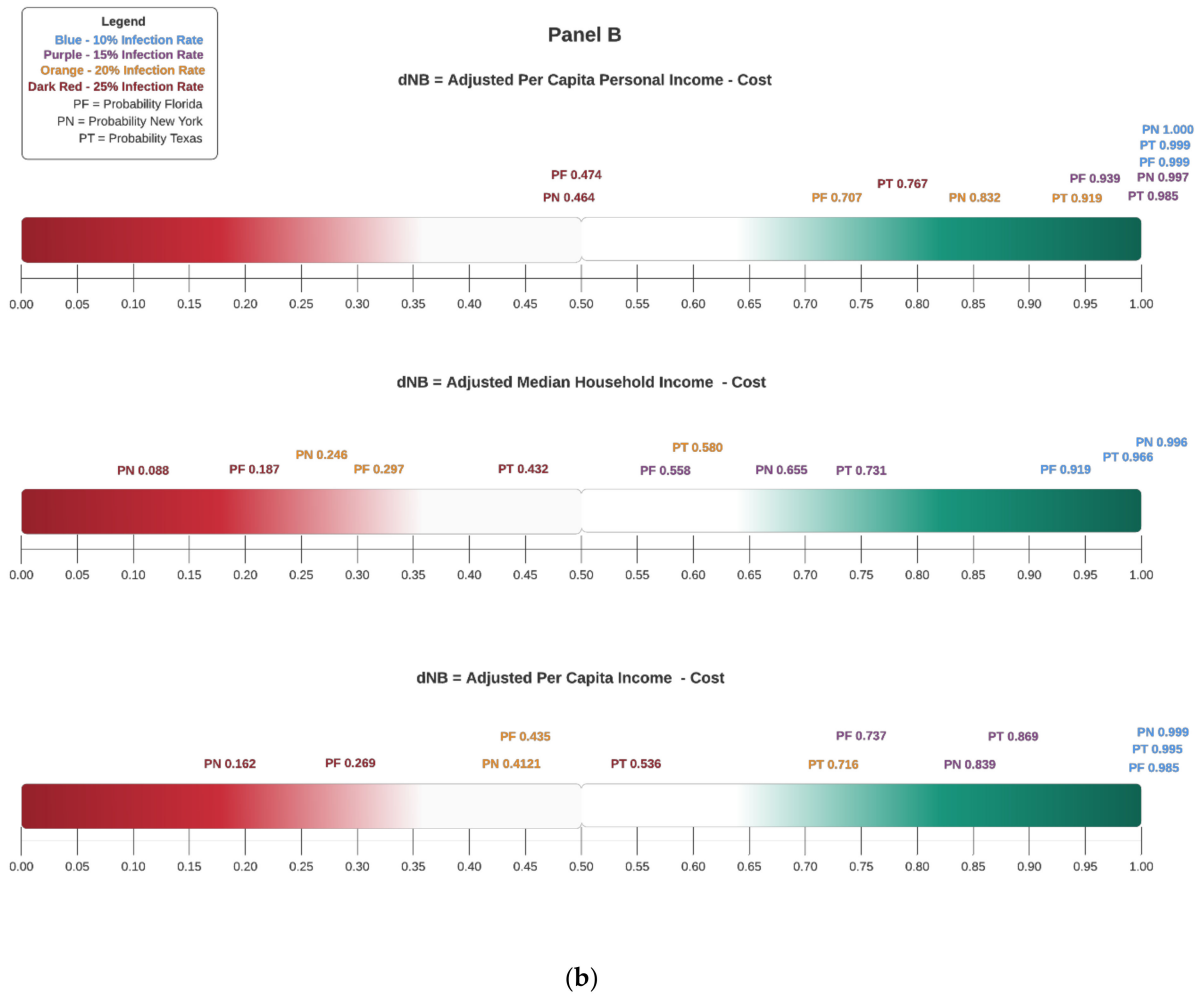
(**a**) Panel A—*P*(ΔNB > 0) with Unadjusted Income Values. (**b**) Panel B—*P*(ΔNB > 0) with Adjusted Income Values.

Consider Figure 7, Panel A. The values shown in this plot are from Table 3, Table 4 and Table 5 and correspond to *P*(ΔNB > 0) for FL, NY, and TX, respectively. The scale is therefore from 0% to 100% (equivalently 0 to 1). Panel A plots the decision rule based on unadjusted incomes, while Panel B plots the decision rule using adjusted incomes. The colour coding makes it clear that, as the threshold probability approaches one, decremental suppression is viable; likewise, when it approaches zero, the opposite is true. Therefore, how large should *T* be for policymakers to decide on decremental suppression? It depends on the individual or group’s attitude to risk; we provide more analysis on this point in the next subsection. Here, suppose for the sake of discussion that decision-makers in all three states decide to base their decision solely on the values shown in the Panel A top row plot; this corresponds to Per Capita Personal Income measure. Clearly, for the scenario corresponding to infection rate 10%, decremental suppression is viable since a positive net benefit can be had with near certainty, likewise for 15%. At 25%, decremental suppression appears most viable for TX. For FL and NY, perhaps, some form of decremental suppression of their respective economies seem warranted. Indeed, that is precisely what FL did while NY was in a complete lockdown. To be clear, in the latter state, infection rates were close 38%. Hence, the probability shown in the plot under the 25% infection rate scenario is an overestimate for NY; in truth, the probability is closer to the lightest red shaded area. The remaining plots in Panels A and B show the Bayesian decision rule graphs for various sensitivity analyses based on different unadjusted and adjusted incomes, respectively. It is interesting to note how the decision rule changes depending on the income measure used when infection rates increase. 

### 4.5. Policy Decision Analyses and Discussion of Threshold Probability T

We can expand on the insights from the plots shown in Figure 7 to more “what if” policy scenarios. In this subsection, we focus on the net benefits of a decremental suppression policy that may range from being minor (e.g., expanding social gathering’s size limit) to major (e.g., removing all suppression measures). Implementation of the policy could cause additional infections, which can be characterized by Table 6 below. With data on the probabilities{*π_jk_*} of incremental infection rates under each policy being absent, we use decision analysis scenarios to set up a government’s policy choice. Note that this type of a decision analysis is similar in spirit to a decision tree model; we prefer to present it in a tabular form since we work with entire predictive distributions from the Bayesian models.

The various assumed probabilities in Table 6 are specific to an incremental infection rate scenario. For the minor policy, the probability of the 10% incremental rate is relatively high at 0.5, exceeding those of the other rates. The moderate policy, however, makes the incremental rate of 15% most likely. The major policy implies high probabilities for the 20% and 25% rates. In summary, the last column of Table 6 highlights that the expected value of infection rate *k* increases with a policy’s extent of decremental suppression.

Varying the assumed probabilities in Table 6 generates different infection rate/policy scenarios given in Table 7 and Table 8, which portray higher expected values of *k* than those in Table 6.

For notational simplicity, let *B_jk_* = ΔNB*_jk_* denote the change in net benefit conditional on infection rate *k* due to policy *j*’s implementation. A standard benefit–cost analysis suggests that policy *j* should be considered if the policy’s implementation results in E(*B_j_*) = *π_k_ π_jk_* E(*B_jk_*) > 0. The goal of a risk-neutral government is to choose j* that results in E(B_j_*) = max{E(*B_j_*)}. For a risk-averse government, its goal is to choose j* so that E(B_j_*) = max{E(*B_j_*)} and *P_j_* = prob(B_j_* > 0) > *T*. 

The threshold *T* reflects a government’s attitude toward risk. Suppose *T* = 0.90. The government is highly risk-averse and only chooses a policy with a positive net benefit that can be attained with almost certainty. Recall from Figure 7 that, when the infection rate was 10%, *P*(ΔNB > 0) was almost one for all three states; i.e., the Bayesian predictive distribution of ΔNB indicated that a positive net benefit could be obtained with near certainty. When *T* declines, the government is willing to take some risk in making its policy choice. 

To make the decision process operational, we assume that a government prepares a look-up table based on the information available on day *d*: (1) observed infection rate, (2) incremental income forecast on day *d*, and (3) incremental cost for fatality and medical treatment. Table 9 below is an example of the look-up table. 

Suppose that a government believes in Scenario 1. If the government is risk-neutral, it selects the minor policy that has the highest net benefit estimate. When the government is risk-averse with *T* = 0.90, it still chooses the minor policy. Using the same line of reasoning for the other two scenarios leads to similar inferences. The overall finding that the minor policy is preferable over the other two policies makes sense because changes in the incremental infection rate does not materially affect the expected incremental income but can greatly magnify the expected incremental cost of fatality and medical treatment.

## 5. Discussion and Conclusions

Referring to Figure 7 and the policy analyses in Section 4.5, a decremental suppression policy decision in Florida, New York, and Texas depends on several factors. First, it would depend on which measure of income one chooses; for instance, the CARES Act package provided by the federal government increases *P*(ΔNB > 0). State local officials should consider whether these aid packages are sustainable if the pandemic is prolonged. Second, regardless of the income measure, should states close all counties or focus on those that have the highest infection rates? Third, are these higher rates amidst vulnerable populations? Fourth, among non-vulnerable groups, does the infection rate mostly affect people with comorbid conditions? The answers to these and related questions must be considered before deciding on decremental suppression. However, our decision rule also cautions against sweeping closures of an entire economy based solely on rising infection rates. It is important to emphasize that Florida officials actually took a very sensible approach by isolating and locking down counties comprising the most vulnerable populations. Thus, they had very low death rates compared with other states. Additionally, they opened parts of the state where infections were not surging. By stating this, we do not discount the relevance and potential dangers of infection; indeed, that is why we use the infection rate as an exogenous factor in our models and sensitivity analyses. In this regard, it is worth recalling eminent 19th century British epidemiologist William Farr’s Law: “The death rate is a fact; anything beyond this is an inference.” 

In addition to the economic value from decremental suppression, epidemiologically, herd immunity can be attained with rising infections. (Using Sweden as a case study, Nobel-laureate Michael Levitt discussed the latter point in the context of COVID-19. https://www.stanforddaily.com/2020/05/04/qa-nobel-laureate-says-COVID-19-curve-could-be-naturally-self-flattening/. https://twitter.com/mlevitt_np2013?lang=en, accessed on 10 July 2020) Note, however, that fatality rate predictions from Equation (2) becoming alarmingly high due to increased infections affects the *P*(ΔNB > 0) estimates. Thus, our decision rule serves as a useful standard of judgment when assessing the economic *and* health aspects of decremental suppression. Indeed, we note that the decisions made by state officials in Florida, New York, and Texas generally coincide with what our model-based output showed to be the preferred decisions. 

**Figure 8:**
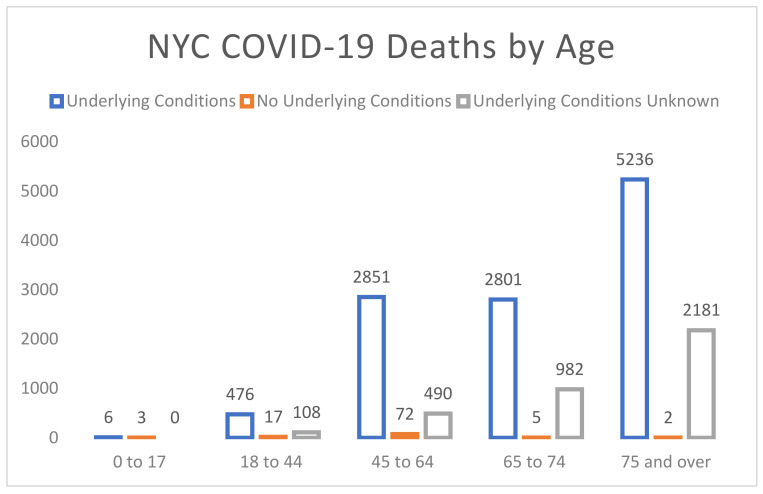
New York City COVID-19 deaths by age as of 12 May 2020. Note: Underlying conditions include diabetes, lung disease, cancer, immunodeficiency, heart disease, hypertension, asthma, kidney disease, GI/liver disease, and obesity.

We now turn our attention to the econometric specifications used in Equations (1) and (2). First, it may be tempting to use cross-sectional regressions to model (1) and (2). We do not recommend this for a few reasons, not the least of which is that the data collection process varies considerably. Some states never closed, while others did so partially. Some closed earlier than others. There was and is considerable variation in social distancing measures adopted by states. However, above all, even within a state, the employment and mortality rates vary considerably. At best, one might want to consider panel data models for states that were affected similarly by the pandemic; for example, New York, New Jersey, Pennsylvania, and Michigan. Rather than panel data models, we suggest using our approach to model employment data by state and fatality data by county. Furthermore, it is useful to consider modelling the latter data to account for age, race, and comorbid health conditions. Indeed, one could replace the continuous mortality model in Equation (2) by a binary logistic regression where the observed response is dead or alive, with age, gender, comorbidities, and race serving as covariates. To this end, consider Figure 8, which shows the number of deaths from the contagion in New York City classified by risk groups. (The data are available at the NYC health data archive. https://www1.nyc.gov/site/doh/COVID/COVID-19-data-archive.page, accessed on 10 July 2020) It is evident that age is a significant factor in the number of deaths. If New York, like Florida, had taken measures to curb infections in the most vulnerable population groups, perhaps its death rates would have been lower. Another striking inference from Figure 8 is the impact of the pandemic on those with comorbid conditions.

There is growing evidence that many of the decisions made by governments based on COVID-19 epidemiological models were highly questionable. A recent *Newsweek* report discussed why lockdowns were ineffective compared with other voluntary measures (https://www.newsweek.com/COVID-lockdowns-have-no-clear-benefit-vs-other-voluntary-measures-international-study-shows-, accessed on 10 July 2020). Additionally, there are at least 30 papers that show lockdowns were generally ineffective (https://inproportion2.talkigy.com/do_lockdowns_work_2021-01-15.html, accessed on 10 July 2020) in sharp contrast to Alveda et al. [26], who argued otherwise. Other NPIs have also come under scrutiny, as have the epidemiological aspects of how the disease spreads. Marks et al. [27] “did not find any association between mask use and reduced risk [of infections]”. Indeed, MacIntyre et al. [28], in a related context, found that cloth masks *increased* infection rates. Governmental lockdowns and other NPI mandates during this pandemic, based on epidemiological models, will be researched and debated (as should be) long after COVID-19 is a distant memory. For now, it becomes even more important to focus on the economic impacts of the pandemic, especially since several pharmaceutical interventions (example, vaccines) are reaching the masses. In our Bayesian decision rule analysis, the observed infection rate serves as a credible proxy to quantify the overall health (due to the pandemic) in each state’s population. That is, our model recognizes the importance of both health effects and economic ones, which is why infection rates appear in both the economic and fatality models. However, to be sure, there is a trade-off between the economic and health impacts. Specifically, based on [26], countries (e.g., France, Germany, Italy, and the U.K.) that sacrificed their economies to save lives have suffered greater economic losses than those that did not (e.g., China, Indonesia, Bulgaria, and Sweden).

Using risk simulations, Rice et al. [29] showed that, no matter the country, the demographic attack profile of this particular virus is that it disproportionately harms those who are already not working and not “producing”, and those with comorbid conditions; see, also, Figure 8 above. The trade-off between health and economic impacts is also relevant while examining death rates. Reports on mortality from COVID-19 glaringly omit the deaths from lockdowns or projected deaths to come over the years from slamming the breaks on society (cancer screenings, mental health, etc.).

One of our conclusions coincide with that of [29], albeit in a somewhat different manner. Our analysis points to a position of compromise—use Bayesian decision analysis to model health effects (via the fatality model) and economic effects (via the employment model). Unlike [29], we do not model age explicitly, but it is implicit in our formulation. We too find that a segmentation approach to decremental suppression could be the best course of action in many instances; see the discussion in Section 4. Additionally, that is precisely what Florida and Texas’s successful segmented strategies accomplished, confirmed by the decision rule in our paper. 

Above all, using our Bayesian methodology, we are able to perform valuable sensitivity analyses that indirectly accounts for various factors that may impact infection rates such as mask wearing, social distancing, etc. Additionally, there are several data issues of particular relevance to the COVID-19 pandemic. For instance, the fraud from the CARES Act is in the millions of dollars. Tracking these by state is impossible. Unemployment insurance scams are also in the millions of dollars. These also vary by state and regions within states. Government pay-outs are eventually paid by taxpayers. It is not clear how and when the taxation changes will be imposed. How all of this plays out in the future remains unknown and uncertain. However, the probabilistic construction explicit in the Bayesian approach encapsulates structural uncertainties and data deficits. This is one of many reasons why decision and risk analysis, based on Bayesian modelling of net benefit per capita, could serve as a useful practical tool during a pandemic, consistent with the discussion in [2].

## Figures and Tables

**Table 1:** Posterior probabilities of models with different number of lags.

	Employment			Fatality Rates		
	FL	NY	TX	FL	NY	TX
1 lag	0	0	0	0	**0.9998**	**0.9999**
2 lags	0	**0.9998**	0	**0.9998**	0.0002	0.0001
3 lags	0.0017	0.0002	0	0.0001	0	0
4 lags	**0.9983**	0	**1**	0	0	0
5 lags	0	0	0	0	0	0
6 lags	0	0	0	0	0	0
7 lags	0	0	0	0	0	0

**Table 2:** Summary statistics for the posterior distributions of the parameters from the Florida employment and fatality rate models.

	Mean	S.D.	95% HPD Interval	
	Panel A: Employment Equation			
*α*_0_	0.6016	0.5895	−0.5086	1.8175
*α*_1_	−5.6685	0.5572	−6.7189	−4.5371
*α*_2_	−0.0511	0.0195	−0.0914	−0.0154
*ϕ*_1_	0.3174	0.0391	0.2357	0.3896
*ϕ*_2_	0.9884	0.0111	0.9643	1.0000
*ϕ*_3_	−0.0559	0.0645	−0.1797	0.0755
*ϕ*_4_	−0.2559	0.0564	−0.3616	−0.1413
*σ* ^2^	0.0239	0.0013	0.0214	0.0265
	Panel B: Fatality Rate Equation			
*γ*_0_	0.0008	0.0057	−0.0102	0.0118
*γ*_1_	0.0467	0.0859	−0.1253	0.2058
*ϕ*_1_	0.8693	0.0628	0.7543	0.9902
*ϕ*_2_	0.0282	0.0261	−0.0238	0.0785
*σ* ^2^	0.0004	0.0001	0.0003	0.0005

**Table 3:** Summary statistics (in dollars) of the ΔNB distributions for Florida.

	(1)	(2)	(3)	(4)
Infection Rate	10%	15%	20%	25%
ΔNB = Personal Income − Cost				
Mean	42,964	27,295	9404	−8564
SD	13,186	20,877	28,086	36,438
2.5 Percentile	17,654	−13,685	−44,606	−80,608
97.5 Percentile	68,504	67,915	63,870	61,562
*P*(ΔNB > 0)	**0.9998**	**0.8996**	**0.6344**	**0.4104**
ΔNB = Adjusted Personal Income − Cost				
Mean	48,666	32,982	15,066	−2914
SD	13,186	20,872	28,085	36,438
2.5 Percentile	23,256	−7920	−38,876	−75,048
97.5 Percentile	74,228	73,731	69,423	67,205
P(ΔNB > 0)	**0.9998**	**0.9394**	**0.7072**	**0.4738**

**Table 4:** Summary statistics (in dollars) of the ΔNB distributions for New York.

	(1)	(2)	(3)	(4)
Infection Rate	10%	15%	20%	25%
ΔNB = Personal Income − Cost				
Mean	59,690	35,824	13,120	−11,278
SD	11,538	17,425	22,596	29,201
2.5 Percentile	37,325	1822	−31,848	−68,426
97.5 Percentile	82,021	70,879	57,320	44,885
*P*(ΔNB > 0)	**1.0000**	**0.9822**	**0.7222**	**0.3500**
ΔNB = Adjusted Personal Income − Cost				
Mean	68,108	44,214	21,482	−2940
SD	11,542	17,427	22,598	29,201
2.5 Percentile	45,781	10,059	−23,415	−60,113
97.5 Percentile	90,523	79,261	65,652	53,279
*P*(ΔNB > 0)	**1.0000**	**0.9974**	**0.8322**	**0.4640**

**Table 5:** Summary statistics (in dollars) of the ΔNB distributions for Texas.

	**(1)**	**(2)**	**(3)**	**(4)**
Infection Rate	10%	15%	20%	25%
ΔNB = Personal Income − Cost				
Mean	44,270	33,702	27,264	16,801
SD	11,651	18,150	23,519	31,388
2.5 Percentile	21,576	−2108	−19,513	−45,996
97.5 Percentile	66,718	69,842	74,158	76,993
*P*(ΔNB > 0)	**0.9998**	**0.9686**	**0.8782**	**0.7046**
ΔNB = Adjusted Personal Income − Cost				
Mean	50,013	39,444	33,005	22,538
SD	11,653	18,150	23,524	31,386
2.5 Percentile	27,291	3547	−13,805	−40,205
97.5 Percentile	72,510	75,494	79,856	82,704
*P*(ΔNB > 0)	**0.9998**	**0.9850**	**0.9188**	**0.7670**

**Table 6:** Scenario 1: probabilities of incremental infection rates by policy type.

Policy Type *j*	Incremental Infection Rate *k* (%)				Expected Value of *k*
	10	15	20	25	
Minor	0.5	0.3	0.1	0.1	14
Moderate	0.2	0.4	0.2	0.2	17
Major	0.1	0.2	0.4	0.3	19.5

**Table 7:** Scenario 2: probabilities of incremental infection rates by policy type.

Policy Type *j*	Incremental Infection Rate *k* (%)				Expected Value of *k*
	10	15	20	25	
Minor	0.3	0.3	0.3	0.1	16
Moderate	0.2	0.3	0.3	0.2	17.5
Major	0.1	0.2	0.4	0.3	19.5

**Table 8:** Scenario 3: probabilities of incremental infection rates by policy type.

Policy Type *j*	Incremental Infection Rate *k* (%)				Expected Value of *k*
	10	15	20	25	
Minor	0.2	0.4	0.3	0.1	16.5
Moderate	0.1	0.2	0.4	0.3	19.5
Major	0.0	0.1	0.4	0.5	22

**Table 9:** E(*B_j_*) and *P_j_* by incremental infection rate scenarios in Florida.

Policy *j*	Scenario 1		Scenario 2		Scenario 3	
	E(*B_j_*)	*P_j_*	E(*B_j_*)	*P_j_*	E(*B_j_*)	*P_j_*
Minor	29,754	0.9988	23,042	0.9726	21,475	0.953
Moderate	19,679	0.9372	17,890	0.913	10,948	0.7496
Major	10,948	0.7496	10,948	0.7496	2209	0.5464

## Data Availability

The data presented in this study are available at the public sources as cited in the main text.

## Supplementary Materials

The following are available online at https://www.mdpi.com/article/10.3390/healthcare9081023/s1. Table S1: Gelman–Rubin statistics for the Florida employment and fatality rate model parameters; Figure S1: Trace plots of parameters of the Florida employment model; Figure S2: Histograms of parameters of the Florida employment model; Figure S3: Trace plots of parameters in the Florida fatality rate model; Figure S4: Histograms of parameters of the Florida fatality rate model.
