# Supplementary material for: A Bayes Decision Rule to Assist Policymakers during a Pandemic
Source: Healthcare (Basel). 2021 Aug 9;9(8):1023. doi: 10.3390/healthcare9081023 (PMC8391194; doi:10.3390/healthcare9081023)
Supplement: Supplementary file 1 [file healthcare-09-01023-s001.zip › healthcare-1285430-supplementary.pdf]

In this Supplementary Material, we provide a set of diagnostic results of the Bayesian estimation models. First, consider the Gelman-Rubin statistic for convergence in the Florida employment and fatality rate models. As shown in Table S1, the convergence diagnostics for all model parameters are less than 1.1, which suggest that all parameters under both MCMC chains have converged. Next, consider the traces plots and histograms of the parameters for both models under the two MCMC chains (in red and blue) which were used in the final analysis. A trace plot is a line that connects consecutive values of the simulated parameters against the iteration number. Thus, a parameter with convergence problems would have a trace plot with sparseness and trends. There are no such patterns in the trace plots. The subsequent histograms of the posterior distributions of the parameters show that the samples from the two chains are generally indistinguishable.

Table S1. Gelman–Rubin statistics for the Florida employment and fatality rate model parameters.

**Employment Equation**		Fatality Rate Equation	
$\boldsymbol{\alpha_0}$	**1.0016**	[illegible]$_0$	1.0007
α_1	1.0053	[illegible]$_1$	1.0009
α_2	1.0029	[illegible]	1.0009
[illegible]	1.0049	[illegible]	1.0015
[illegible]	1.0542	σ^2	1.0000
[illegible]	1.0040		
[illegible]	1.0013		
σ^2	1.0002		

Note. Converged if the Gelman–Rubin statistic < 1.1.

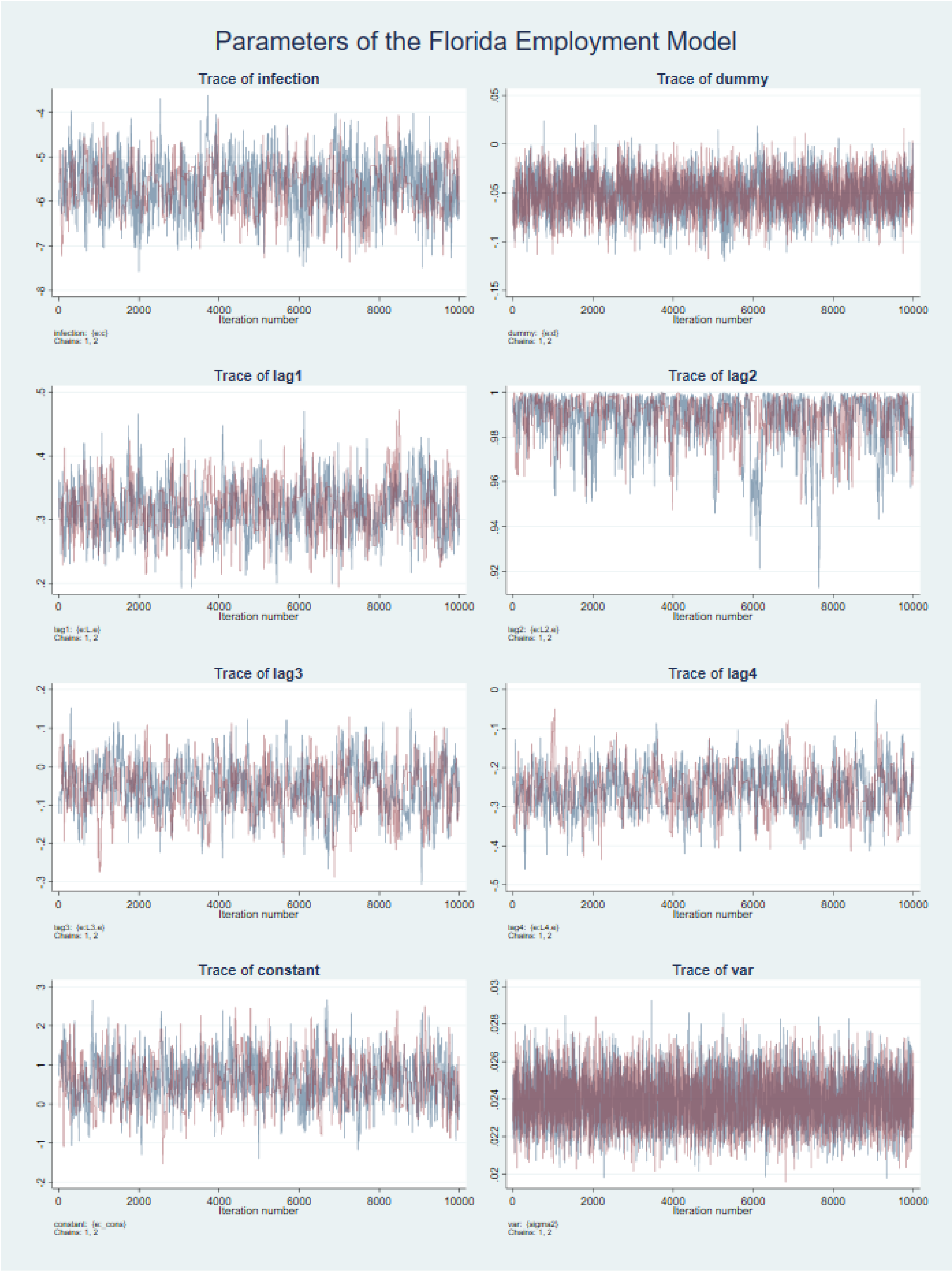

Figure S1. Trace plots of parameters of the Florida employment model

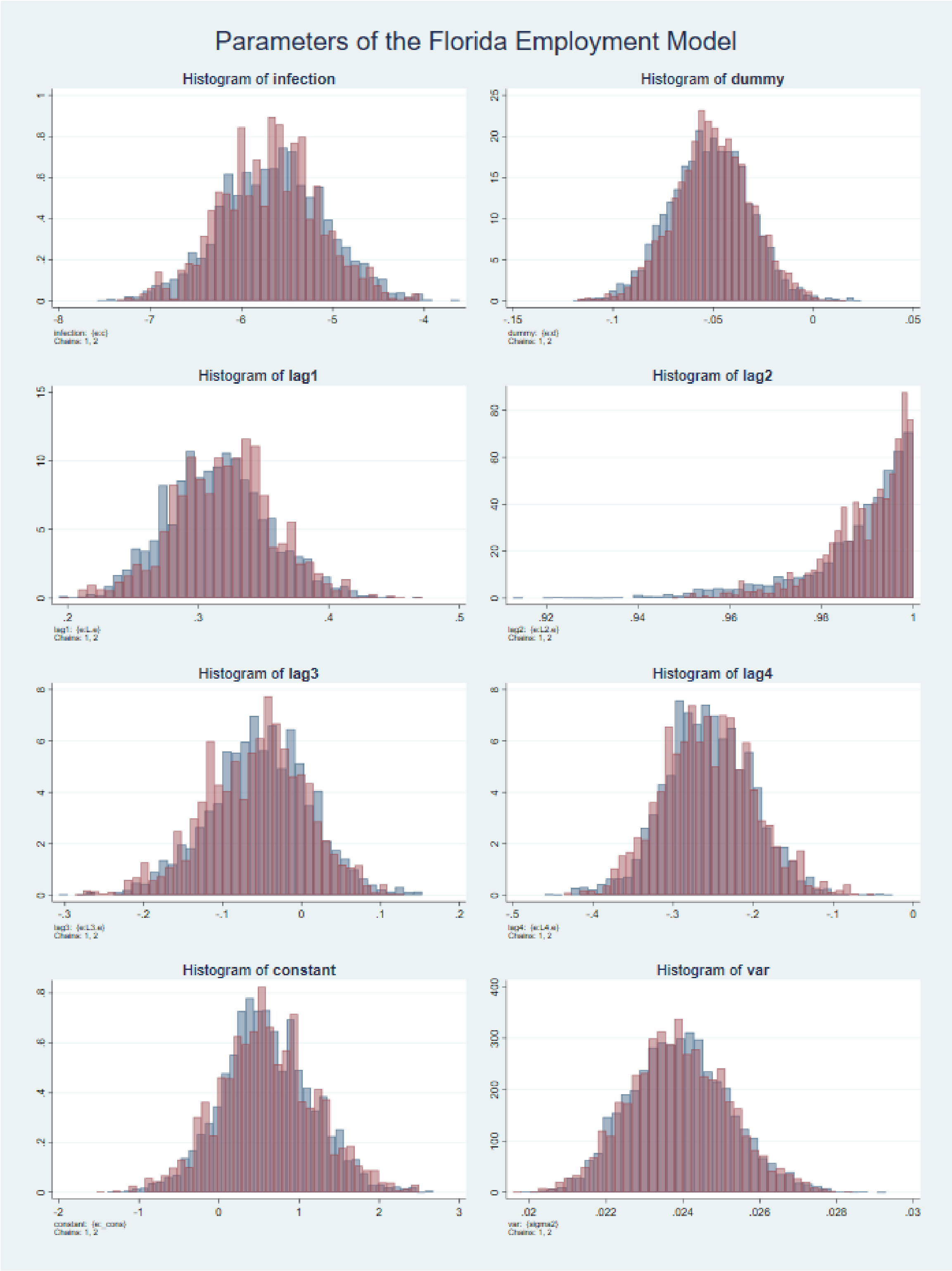

Figure S2. Histograms of parameters of the Florida employment model

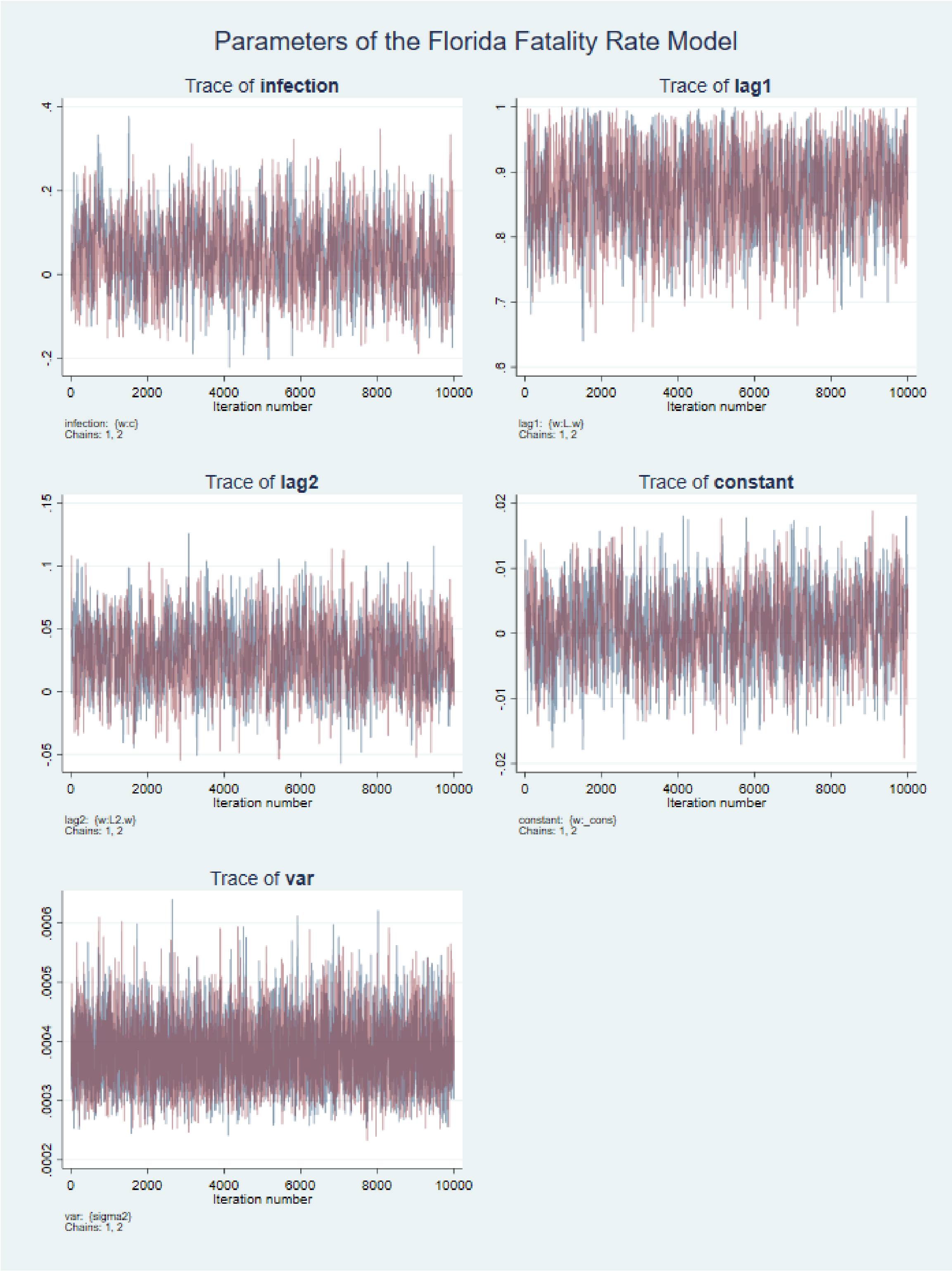

Figure S3. Trace plots of parameters in the Florida fatality rate model

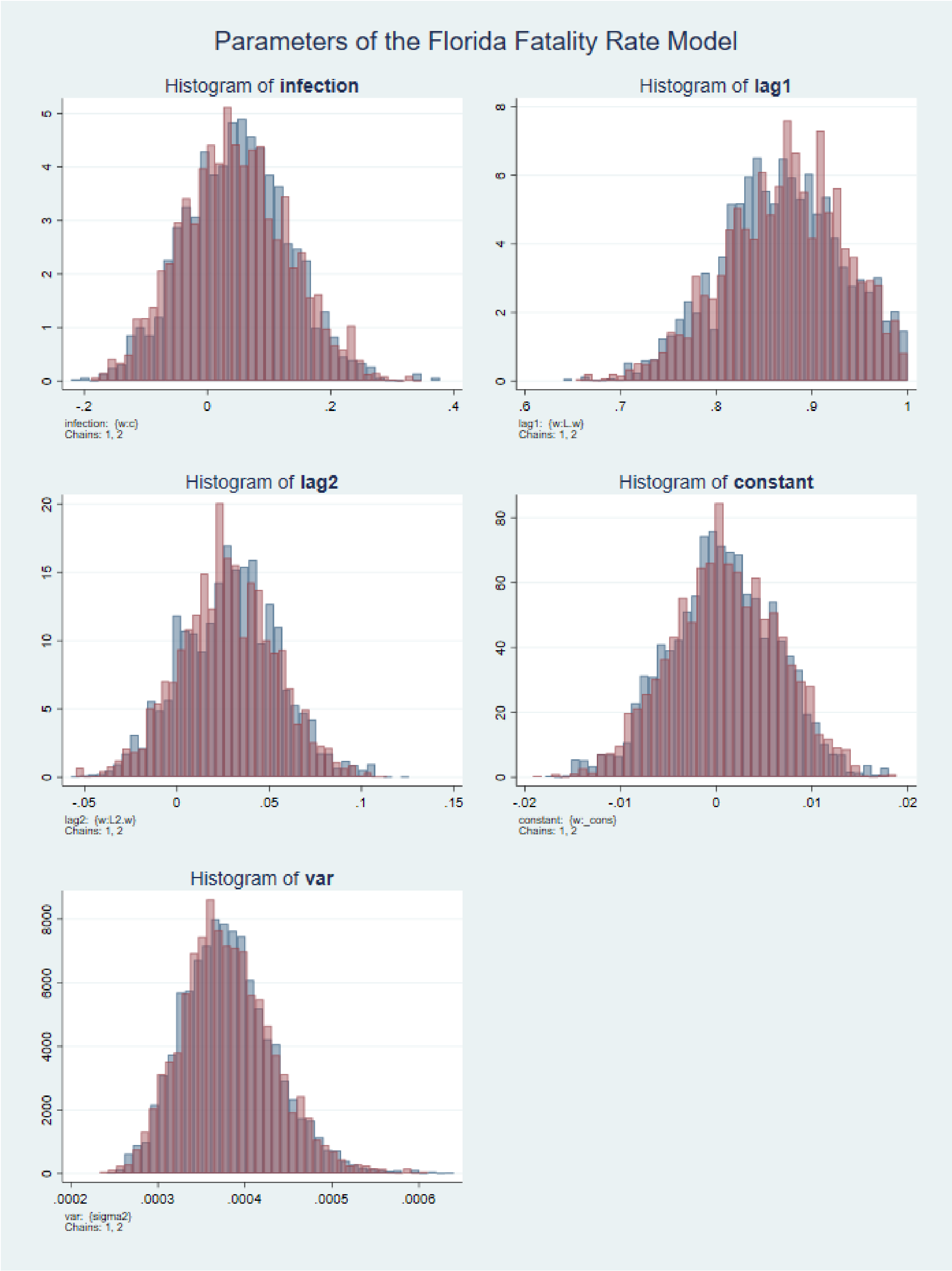

Figure S4. Histograms of parameters of the Florida fatality rate model
